# Endometriosis: Cellular and Molecular Mechanisms Leading to Fibrosis

**DOI:** 10.1007/s43032-022-01083-x

**Published:** 2022-10-26

**Authors:** Jose Manuel Garcia Garcia, Valentina Vannuzzi, Chiara Donati, Caterina Bernacchioni, Paola Bruni, Felice Petraglia

**Affiliations:** Obstetrics and Gynecology and Molecular Biology, Department of Experimental and Clinical Biomedical Sciences “M. Serio, ” University of Florence, Florence, Italy

**Keywords:** Endometriosis, Endometrioma, Deep infiltrating endometriosis, Fibrosis, TGF-β, S1P

## Abstract

Endometriosis is a chronic inflammatory condition affecting women of reproductive age. A relevant feature of endometriosis is the presence of fibrotic tissue inside and around the lesions, thus contributing to the classic endometriosis-related symptoms, pain, and infertility. The molecular mechanisms responsible for the development of fibrosis in endometriosis are not yet defined. The present review aimed to examine the biological mechanisms and signalling pathways involved in fibrogenesis of endometriotic lesions, highlighting the difference between deep infiltrating and ovarian endometriosis. The main cell types involved in the development of fibrosis are platelets, myofibroblasts, macrophages, and sensory nerve fibers. Members of the transforming growth factor (TGF) -β family, as well as the receptor Notch, or the bioactive sphingolipid sphingosine 1-phosphate (S1P), play a role in the development of tissue fibrosis, resulting in their metabolism and/or their signalling pathways altered in endometriotic lesions. It is relevant the knowledge of the molecular mechanisms that guide and support fibrosis in endometriosis, to identify new drug targets and provide new therapeutic approaches to patients.

## Introduction

Endometriosis is a chronic inflammatory and sex steroid hormone-dependent gynaecologic disease, characterized by endometrial cell proliferation outside the uterine cavity [1, 2], diagnosed in 6–10% of all reproductive-age women [3]. The most common symptoms of endometriosis are menstruation-related pain (dysmenorrhea, dyspareunia, dysuria, dyschezia) and infertility, significantly affecting the quality of life [4]. Endometriotic lesions are classified into 3 phenotypes: superficial peritoneal (SUP), ovarian endometrioma (OMA), and deep infiltrating endometriosis (DIE)[1]. They are characterized by endometrial cell proliferation and invasion of peritoneum and oxidative stress, associated with inflammation and neuroangiogenesis [5, 6]. Furthermore, a fibrotic reaction is described in different degrees according to the location of endometriosis and may contribute to chronic pain and infertility. The aim of the present review is to show the mechanisms related to the establishment and progression of fibrosis in endometriosis, emphasizing the molecular aspects involved in the different localizations of the disease, in order to facilitate the development of new therapeutic approaches.

### Mechanisms of Fibrosis

The formation of fibrotic tissue is characterized by the excessive accumulation of components of the extracellular matrix (ECM), inside and around inflamed or damaged tissue, and represents an usual and significant phase of tissue repair in all organs [7]. The biological process of fibrosis requires the involvement of activated platelets, macrophages, and myofibroblasts, which in turn, contribute to high levels of transforming growth factor (TGF)-β, and the deposition of collagen [7, 8]. Indeed, subsequent to a tissue injury, platelets are activated and aggregate, leading to the formation of the fibrin clot and to the release of TGF-β. The recruitment of neutrophils and monocytes/macrophages then occurs with amplification of the acute inflammatory response, followed by the proliferation and activation of the effector cells to re-establish the barrier function and to induce neoangiogenesis. Finally, the myofibroblasts derived from different progenitor populations are recruited, representing the principal responsible for the deposition, remodelling, organization, and maturation of scar tissue [9, 10, 11]. When the damage is minor or non-repetitive, wound healing is efficient, but when the injury is repetitive or the resolution is improper, components of the ECM continue to accumulate, leading to permanent scarring, disruption of tissue architecture, and organ dysfunction [8].

Myofibroblasts can be originated from different sources, including tissue resident fibroblasts, epithelial and endothelial cells undergoing epithelial/endothelial-to-mesenchymal transition (EMT/EndMT), as well as from circulating fibroblast-like cells named fibrocytes, originated from bone-marrow stem cells [12]. Myofibroblasts are characterized by the expression and incorporation of *α* smooth muscle actin (αSMA) into stress fiber-like microfilaments bundles, which are crucial for promoting the specific function of these cells to contract the ECM [12].

TGF-β is the main profibrotic cytokine, which acts on fibroblasts and myofibroblasts by inducing their proliferation, migration, and matrix production [13]. There are three TGF-β isoforms (TGF-β1, 2, and 3): TGF-β1 is crucial for the development of fibrosis and inflammation. TGF-β1 exerts its biological effects via the heterodimerization of two transmembrane receptors, TGF-β receptor Type I (TGF-βRI) and Type II (TGF-βRII) [14], and it can induce both canonical (Smad-dependent) and non-canonical (non-Smad-dependent) signalling pathways, which result in activation of myofibroblasts, increase of ECM production, and inhibition of ECM degradation [15, 16]. NR4A1 (also known as TR3, Nur77m, or NGF-IB) is a member of the steroid receptor/thyroid hormone superfamily and is an important endogenous inhibitor of TGF-β signalling [17]. In physiological wound healing, the temporary upregulation of TGF-β induces NR4A1 expression creating a negative feedback loop, while the persistent activation of TGF-β signalling in fibrotic diseases uses protein kinase B (PKB), also known as AKT, and histone deacetylase-dependent mechanisms to inhibit NR4A1 expression and activation [17].

Activin A belongs to the TGF-β superfamily and is involved in multiple physiological and pathological processes. Activin A signals by binding to one of the two types II receptors on the cell surface (ActRIIA or ActRIIB), which leads to the enrolment of type I receptors ActRIB (ALK4), and to the activation of Smad-dependent or Smad-independent signalling pathways [18].

Extensive cross-talk between the TGF-β/Smad signalling cascade and the bioactive lipid sphingosine 1-phosphate (S1P) has been reported [19]. Moreover, TGF-β strongly induces the expression and activity of sphingosine kinase (SK) [20], the enzyme responsible for the generation of S1P. Several studies have shown that S1P plays a crucial role in tissue fibrosis [21] regulating different aspects, including the permeability of the vascular barrier, the recruitment of inflammatory cells, and the proliferation and differentiation of myofibroblasts, through the transactivation of the TGF-β signalling pathway [22]. S1P synthesis occurs by two isoforms of SK, SK1, and SK2, through adenosine triphosphate (ATP)-dependent phosphorylation of sphingosine, whereas its degradation occurs via two different pathways: the reversible dephosphorylation catalyzed by specific phosphatases (SGPP1 and SGPP2) and the irreversible cleavage by S1P lyase (SGPL). S1P can act either as an intracellular messenger or as a ligand for its membrane receptors named S1P receptors (S1P_1-5_) after its release in the extracellular medium by specific transporters [23].

### Endometriosis-Related Fibrosis

The fibrosis mediators described above play a key role in the onset and progression of fibrosis in all types of endometriosis. The specific mechanisms for each lesion will be explained in detail below.

#### Ovarian Endometriosis (OMA)

Histologically, it is well known that fibrosis is present in the ovarian cyst wall, since OMA wall appears as endometriotic tissue surrounded by scar elements and a rim of ovarian tissue [24]. Positive immunostaining for αSMA antibody has been shown in all OMA lesions [25, 26]. In fact, OMA capsules are characterized by fibrotic tissue more frequently than non-endometriotic cysts. Kitajima et al.[27] observed a frequency of fibrosis, identified by Masson’s trichrome stain, equal to 80% in the cortical tissue of ovaries with OMA and 27% in the healthy ovaries, contralateral to the lesion. They also observed concomitant loss of cortex-specific stroma in 55% of cortical samples from ovaries with OMA, but in none of those from contralateral healthy ovaries, in accordance with the finding that the excised ovarian tissue along with the OMA wall are related with the presence of pericystic fibrosis [24].

**Fig. 1:**
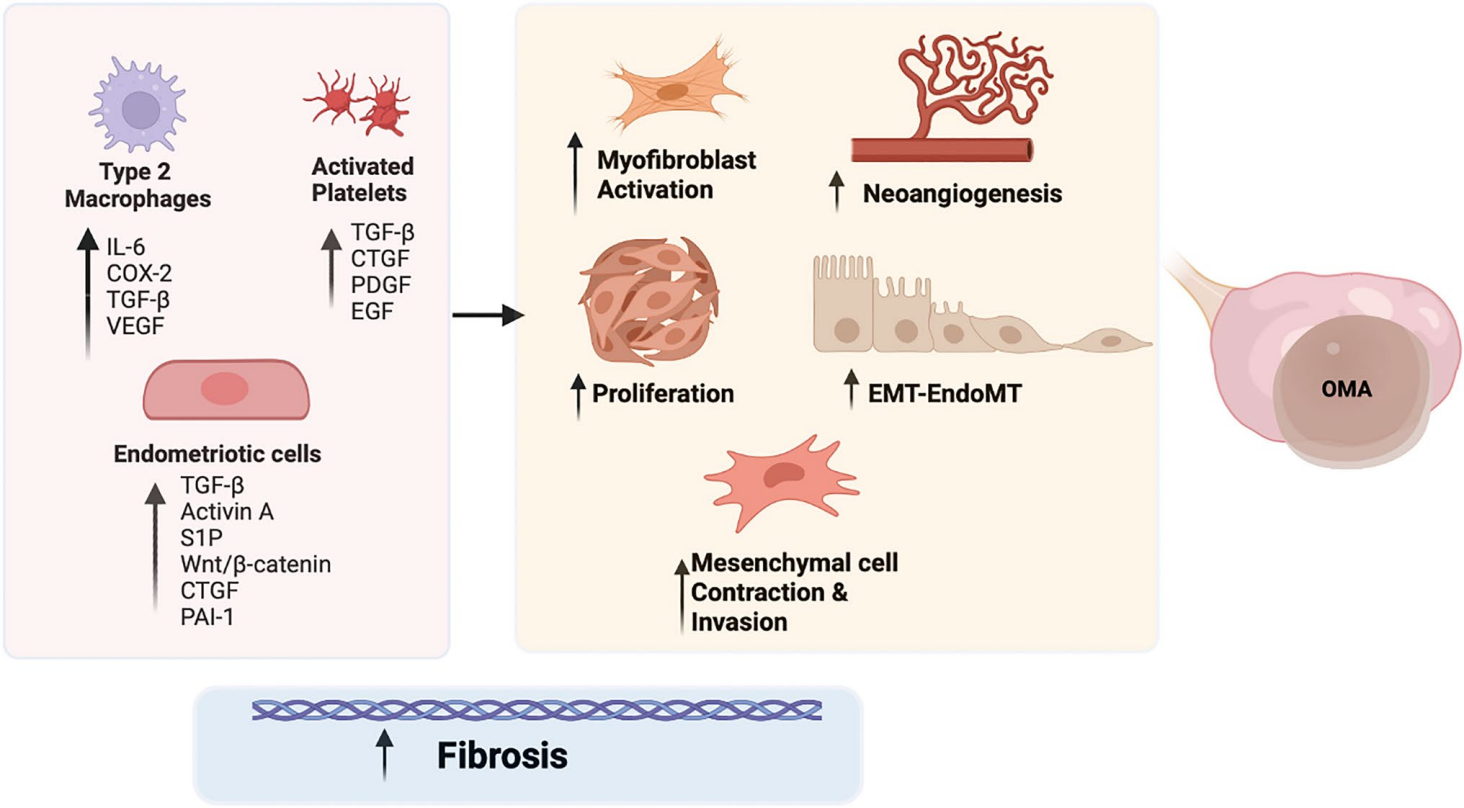
Main cellular types and molecules involved in the development of ovarian endometriosis − related fibrosis; COX-2, cyclooxygenase 2; CTGF, connective tissue growth factor; EGF: epithelial growth factor; EMT, epithelial to mesenchymal transition; EndoMT, endothelial to mesenchymal transition; IL-6, interleukin 6; OMA, ovarian endometrioma; S1P, sphingosine-1-phosphate; PAI-1, plasminogen activator inhibitor-1; PDGF, platelets derived growth factor; TGF-β, transforming growth factor β; VEGF, vascular endothelial growth factor; Wnt, wingless-related integration site

Many factors have been suggested to be implicated in the fibrogenesis of OMA (Fig. 1). An important cellular process closely related to the onset of tissue fibrosis is the EndMT that is responsible for making endothelial cells able to proliferate and acquire de novo the capacity to contract, migrate, invade, and produce collagen. EndMT occurs with a higher incidence in OMA than in DIE, hypothesizing that OMA is more angiogenic than DIE and the improved microvascularity may provide an abundant substrate for EndoMT [28]. Platelets play a critical role in endometriosis-associated fibrogenesis: activated platelets within lesions release numerous growth factors, cytokines, and chemokines such as TGF-β1, platelet-derived growth factor (PDGF), epidermal growth factor (EGF), and connective tissue growth factor (CTGF). This microenviroment, produced by the activation of platelets, represents an advantageous environment for EMT and EndoMT and may directly promote fibrogenesis [28] (Fig. 1).

A critical role of TGF-β in the beginning and progression of fibrosis in OMA has been shown [29, 30, 31, 32, 33]. Endometriotic cells can synthesize TGF-β1 which accumulates in the surrounding ovarian tissue, disorganizing ECM and promoting fibrosis around OMA (Fig. 1). The TGF-β1/Smad signalling pathway improves fibrosis and adhesion of ovarian surrounding tissue [32], suggesting possible TGF-β1-targeting therapeutic strategies. A persistent stimulation of endometriotic tissues with TGF-β1 was able to reduce NR4A1 activity through AKT-dependent phosphorylation, promoting fibrogenesis in OMA [34] and the treatment with Csn-B, an NR4A1 agonist, markedly decreased the expression of fibrotic markers in vitro and inhibited fibrogenesis in urine endometriosis models, suggesting a new potential target for the treatment of this disease [34]. However, it is important to note that because endometriotic lesions of nude mice developed under the skin rather than in the ovary, the mouse model has severe limits compared to human ovarian endometriosis.

Activin βA subunit is strongly expressed in OMA [35, 36, 37] and Activin A concentration in the cystic fluid is higher than in peritoneal fluid in patients with OMA, indicating that this type of lesion highly express this cytokine [35]. Activin A facilitates the invasion of endometrial stromal and epithelial cells in an in vitro model of peritoneum [38], suggesting its possible involvement in the pathogenesis of endometriosis. Activin A also plays a critical role in the differentiation of endometrial stem cells towards the myofibroblast phenotype [39]. Especially, in endometrial stem cells, Activin A leads to the production of CTGF, a key fibrotic marker in fibrotic disorders, such as endometriosis and intrauterine adhesions, through STAT3-dependent Smad signalling [39] (Fig. 1). Finally, in a mouse model of endometriosis, an antibody against activin A significantly inhibited the excessive collagen deposition and the expression levels of collagen I (Col-I), αSMA, and CTGF in ectopic lesions, supplying the experimental basis to treat endometriosis-related fibrosis through the manipulation of activin A signalling.

CTGF is a specific target molecule of miR-214 [40]. MiR-214, one of the downregulated miRNAs in human endometriotic cyst stromal cells (ECSC), is known for having fibrotic suppressor roles, including inhibition of fibroblast proliferation, of collagen synthesis, and of the EMT process [41]. Indeed, an enhanced production of miR-214 decreased the expression of CTGF and Col-I in stromal and endometrial epithelial cells of OMA, in response to stimuli that induce fibrosis [42]. With this finding in mind, it will be interesting to investigate the diagnostic and therapeutic employment of miR-214 in endometriosis patients.

The treatment with the conditioned medium of endometriotic mesenchymal stem cells (Ecto-MSC) significantly increased the expression of genes involved in fibrogenesis in ECSCs via the Wnt/β-catenin pathway by paracrine production of TGF-β1 and Wnt1 [43], activating proliferation, invasion, and contraction of the collagen gel, thus supporting that Ecto-MSCs play a key role in the development of OMA (Fig. 1).

S1P which is abundant in blood constituents, principally in erythrocytes and platelets, has an important role in the proliferation of endometriotic cells [44]. High levels of S1P in fact have been found in the peritoneal environment of women suffering of endometriosis, showing the capability to induce differentiation of macrophages into CD163 + M2 type, which secretes pro-inflammatory cytokines such as IL-6, COX-2, and TGF-β contributing to the inflammation and fibrosis underlying these lesions [45].

Dysregulation of metabolism and signalling of S1P in endometriosis have been recently characterized [15]. In particular, an increase in S1P_1_, S1P_3_ and S1P_5_ mRNA levels, which could activate cell proliferation and migration, has been discovered in OMA, as previously reported in the fibrogenesis of numerous other different tissues [46]. Moreover, S1P is part of TGF-β1-induced fibrosis in an in vitro EMT model of uterine adenocarcinoma cells. Indeed, when SKs and S1P_2/3_ were inhibited, the fibrotic activity of TGF-β1 was blocked. Finally, S1P was been shown to enhance fibrotic markers on its own, via S1P_1/2/3_ [15]. These findings suggest the hypothesis that S1P may be used as a novel biomarker for endometriosis and its signalling targeted for therapeutic purposes [15]. Additionally, altered levels of sphingomyelin in the peritoneal fluid of OMA patients have been observed [47], supporting a crucial role of bioactive sphingolipids in endometriosis. Gene expression profiling for OMA showed altered expression of genes involved in sphingolipid metabolism, with an over-expression of alkaline sphingomyelinase, ceramidase, and SK1 and a downregulation of SGPP1 [48].

The level of plasminogen activator inhibitor 1 (PAI‐1) is significantly higher in chocolate cyst fluid than in other benign cysts [49] (Fig. 1). PAI-1 is a member of the serine protease inhibitor gene family and the major physiologic inhibitor of the serine proteases, urokinase-type plasminogen activator, and tissue-type plasminogen activator (uPA/tPA). Under normal physiologic conditions, PAI-1 regulates the activities of uPA/tPA/plasmin/MMP proteolytic activities preserving the tissue homeostasis. In fibrotic tissues, the level of PAI-1 is elevated, contributing to excessive accumulation of collagen and other ECM proteins in the wound area, preventing tissue proteolytic activities and thus preserving scarring [50].

An interesting observation is that older ovarian cysts, with more episodes of cyclical bleeding, contain chocolate fluid which is higher in density, viscosity, and iron content and display a higher fibrotic composition than younger ovarian cyst, highlighting a difference within the same type of lesion [51]. Finally, older lesions and higher staining levels of αSMA positively correlate with more extensive fibrotic content, while the lesion size and the E-cadherin staining are negatively associated with the grade of fibrosis suggesting that OMA could be a progressive disease [52].

#### Deep Infiltrating Endometriosis (DIE)

Patients with DIE have pain and infertility, associated with smooth muscle proliferation and fibrosis [53]. Histological patterns of these implants include well-differentiated glands, pure stromal, mixed differentiated, and pure undifferentiated cells [54, 55]. Fibrosis is part of the injury, and progesterone receptors are present not only in glands and stroma but also in smooth muscle cells and fibrotic tissue adjacent the DIE lesions [56]. Epithelial cells and endometrial stroma in ectopic sites, such as rectum, vagina, or the peritoneum imply survival and proliferation in a different microenvironment.

**Fig. 2:**
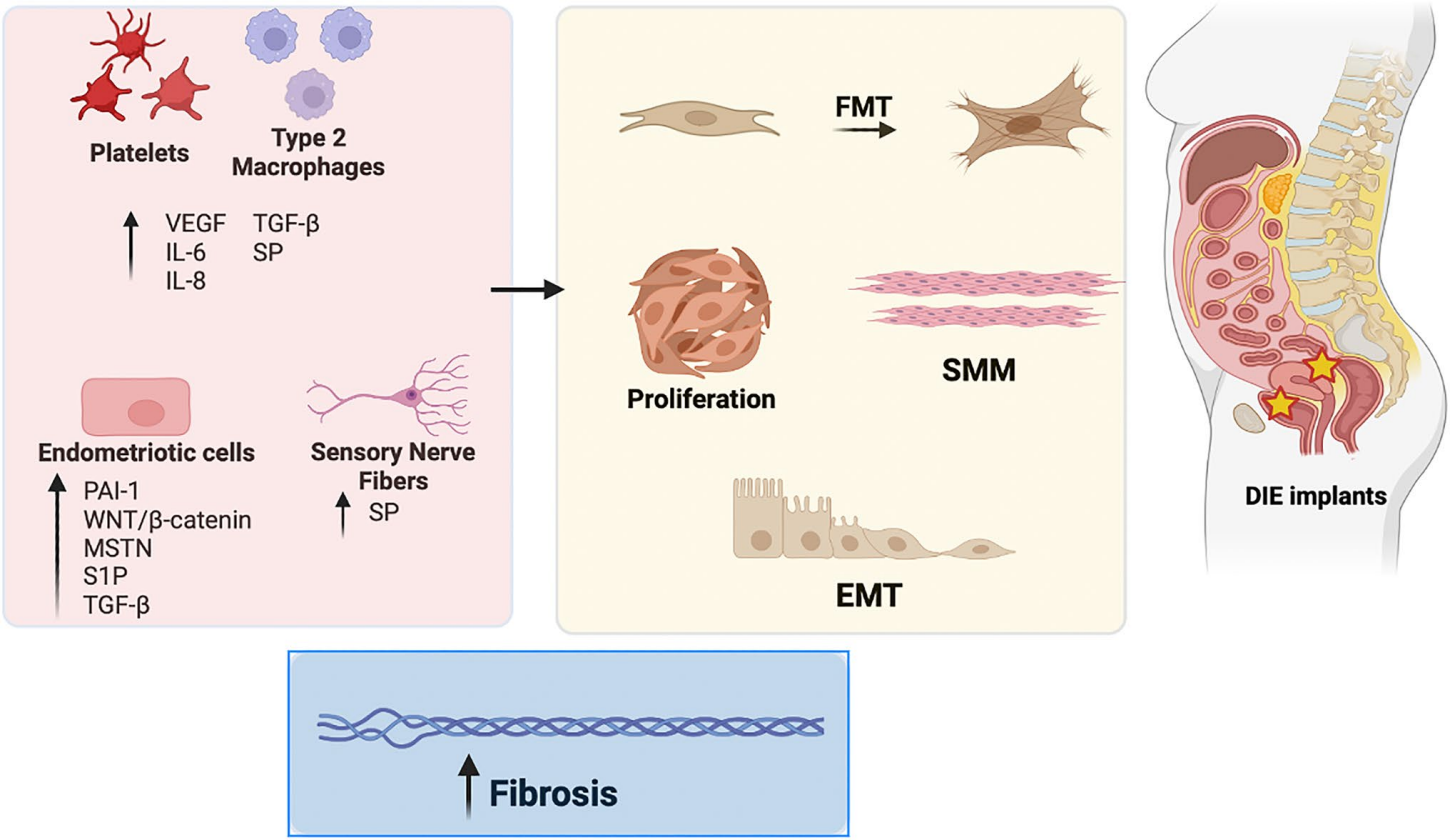
Principal cellular types and molecules implicated in the development of DIE-related fibrosis. DIE, deep infiltrating endometriosis; EMT, epithelial to mesenchymal transition; FMT, fibroblast to myofibroblast transition; IL-6, interleukin 6; IL-8, interleukin 8; MSTN, myostatin; SMM, smooth muscle metaplasia; SP, substance P; S1P, sphingosine-1-phosphate; PAI-1, plasminogen activator inhibitor-1; TGF-β, transforming growth factor β; VEGF, vascular endothelial growth factor, Wnt, wingless-related integration site

Endometriotic lesions undergo recurrent tissue injury and repair. This process is an outcome of TGF-β1, vascular endothelial growth factor (VEGF), IL-6 and IL-8 secretion by platelets and macrophages, as well as endometriotic production of PAI-1, and changes in the expression of MMPs and tissue inhibitors of MMPs which together, with local nerve fibers, participates to EMT, fibroblast to myofibroblast transformation (FMT), and smooth muscle metaplasia (SMM) [53, 57] (Fig. 2).

Myostatin (MSTN), a TGF-β family component which plays a key role in cell proliferation and differentiation, is highly expressed in endometriotic lesions, suggesting MSTN involvement in this pathology [58] (Fig. 2). In particular, the mRNA levels of MSTN and its receptors (ALK 5 and ActRIIB) were significantly higher in DIE than in OMA and control endometrium [58]. When MSTN bind its receptors ALK5 and ActRIIB, Smad 2/3 gets involved in promoting the proliferation of fibroblasts, their differentiation into myofibroblasts, and expression of ECM proteins, thus contributing to fibrogenesis [59]. Furthermore, MSTN shares with TGF-β1 a positive feedback loop, with TGF-β1 enhancing MSTN expression, and, conversely, MSTN inducing TGF-β1 secretion [59].

DIE lesions also exhibit increased SK1, S1P_3_ and S1P_5_ mRNA levels and the expression of a specific S1P transporter, Spns2, responsible for the extracellular release of S1P, is altered only in DIE, further highlighting the dysregulation of S1P signalling in the different forms of endometriosis [15] (Fig. 2).

Peritoneal oxidation protein products are augmented in DIE. Reactive oxygen species (ROS) with metalloprotease ADAM17 induce the release and transport of Notch intracellular domain (NICD), which in turn translocates into the nucleus and are directly involved in the transcriptional regulation of nuclear target genes that result in fibrotic processes, as proven by the expression of Col-I and αSMA in ectopic lesions of patients with DIE [60].

The progression of DIE lesions is also influenced by the Wnt/β-catenin pathway activation, increasing stromal cell proliferation, migration, and contraction of collagen (Fig. 2). Wnt signalling regulates expression of fibrotic marker genes, including CTGF, Col-I, αSMA, and fibronectin [61]. Forkhead box protein P1 (FOXP1), is a transcription factor that acts as an activator of Wnt signalling by promoting β-catenin acetylation and its expression is increased in stromal cells of DIE [61]. Molecular and cellular pathways implicated in fibrogenesis are inhibited by targeting the Wnt/β-catenin signaling in endometrial and endometriotic stromal cells in vitro [62].

Fibroblast proliferation is vital for physiologic wound healing and a dysregulation of this process may lead to excessive scarring and fibrosis. Even if it is known that endometriotic lesions and wound healing share some similarities, further research is required to understand how endometriotic stromal cells (ESC) can proliferate and persist in a fibrotic environment. Integrins, such as α1β1 and α2β1, are main Col-1 receptor [63] ligands and activate cell survival pathways, including those which imply phosphatidylinositol 3-kinase (PI3K) and the serine/threonine kinase AKT, together with the mitogen-activated protein kinase/extracellular regulated kinase (MAPK/ERK) [64, 65]. The study conducted by Matsuzaki et al. showed an increased phosphorylation of AKT and ERK in ESCs than in endometrial stromal cells. Furthermore, the in vitro results proposed that aberrant activation of both signalling pathways might support progression of DIE lesions by enhancing the survival in a fibrotic environment [65].

DIE lesions also present some tumor-like characteristics. Indeed, six cancer driver genes, *TP53*, *PTEN*, *ARID1A*, *PIK3CA*, *KRAS*, and *PPP2R1A*, have been reported to be mutated in endometriosis [66]. *TP53*, *PTEN*, and *ARID1A* mutations are inactivated, while *PIK3CA*, *KRAS*, and *PPP2R1A* are activated. All these genes play an important role in fibrogenesis; for example *TP53* deletion in fibroblasts is found to diminish senescence and to increase TGF-β1 expression, leading to enhanced activated fibroblasts, more extracellular matrix deposition, reduced immune surveillance, and increased fibrosis [66]. Even if a certain profile of somatic cancer driver mutations is found in DIE, its functional relevance is an open theme, and it can be supposed that they could promote tumor-like invasion of endometriotic tissue. Alternatively, these mutations are both cause and consequence of extensive fibrosis around deep nodules. Further research in animal models that could mimic both anatomical presentation and somatic mutations, will be necessary to answer these questions.

Substance P (SP), a member of the tachykinin neuropeptide family widely distributed in central and peripheral nervous system, and produced also by lymphocytes, macrophages, neutrophils, and dendritic cells, has a role in pain, inflammation, and fibrosis. In fact, SP is involved in the fibrosis of many organs (heart, intestine, kidney, lung) and in endometriosis-associated fibrosis [67, 68, 69] (Fig. 2). It is well demonstrated that sensory nerve-derived neuropeptides facilitate lesion fibrogenesis through EMT, FMT, and SMM. Indeed, SP may mobilize mesenchymal stem cells [70] and endothelial progenitor cells (EPC) [71] in bone marrow, inducing migration to damaged peripheral tissues where they participate in tissue regeneration. SP can also induce M2 polarization of inflammatory macrophages that are involved in tissue repair and fibrogenesis processes [33, 72], proposing its role as a treatment to regulate multiple tissue inflammation-related diseases and acute injury. Finally, SP induces PAI-1 and simultaneously reduces tPA levels, resulting in decreased fibrinolysis as well as in the formation of postoperative adhesions and thus exacerbating fibrosis [73, 74].

### Clinical Implications

Cellular and molecular pathways at the basis of fibrosis may represent a target for treating women with endometriosis. Therefore, studies on new possible drugs are in development (Table 1).

**Table 1:** New possible drugs

Pathways	Target	Pathophysiology	Medication	Type/class
S1P signalling	S1P receptor agonist	Proliferation, migration, apoptosis, inflammation and fibrogenesis	Fingolimod	Pro-drug
CBP/β‐catenin	CBP/β‐catenin complex	Proliferation, migration, apoptosis, and fibrogenesis	C-82	Nonhormone, β‐catenin inhibitor
CBP/β‐catenin	CBP/β‐catenin complex	Proliferation, migration, apoptosis, and fibrogenesis	ICG-001	Nonhormone, β‐catenin inhibitor
Wnt/β‐catenin	Tcf/β‐catenin complex	Proliferation, migration, invasion & fibrogenesis	PKF115‐584/CGP049090	Natural product
TGF‐β1‐stimulated/activation of MAPK and Smad pathway & E2/ER/VEGF	/	Proliferation, migration, invasion & fibrogenesis	EGCG	Natural product, catechin
RLX-2	Decreased the phosphorylation of p38MAPK in ESCs	Fibrogenesis and inflammation	Relaxin 2	Produced by the corpus luteum and placenta

Epigallocatechin-3-gallate (EGCG) is one of the principal polyphenols present in green tea, and it has been demonstrated that may lead to inhibition of cell proliferation and angiogenesis, decreasing the size of endometriotic implants in animal models [75]. EGCG can also reduce collagen gel contraction of endometriotic stromal cells, a key aspect of fibrogenesis. EGCG treatment significantly decreases the expression of genes known to be implicated in fibrotic process in endometriotic stromal cells. Moreover, animal experiments displayed that EGCG treatment prevents the development of fibrosis in endometriosis. Its role may be involved in VEGF/VEGFR2 signalling and TGF‐β1‐stimulated/MAPK and Smad pathway [76].

Another molecule, named PKF115‐584/CGP049090, which is an antagonist of Wnt/β-catenin signalling pathway, may have a role in preventing or regressing fibrosis in endometriosis. This experimental drug is a small-molecule antagonist of the T-cell factor Tcf/β-catenin complex that disrupt the critical protein–protein interaction between β-catenin and Tcf and also may decrease TGF-β1 induced fibrotic markers expression in endometrial and endometriotic stromal cells [62]. However, a serious concern in targeting Wnt/β-catenin pathway is the effect this may have in physiological cell renewal: additional studies are needed to assess side effects of in vivo use of the Wnt/β-catenin antagonists in endometriosis.

FTY720 (fingolimod, GilenyaR, 2-amino-2[2-(4-octylphenyl)ethyl]-1,3-propanediol) is a structural analogue of S1P derived from myriocin, which acts as a pro-drug that must be phosphorylated by SK2 to be transformed into the active metabolite fingolimod-P. The latter is a potent but non-selective S1P receptor agonist, binding with similar affinity as S1P to S1P_1_, S1P_3_, S1P_4_ and S1P_5_, but not S1P_2_ [77]. Fingolimod is the first FDA-approved oral drug for treating relapsing–remitting multiple sclerosis [78]. In addition to its immune-modulating effect, fingolimod has a number of additional useful actions, including anti-inflammatory, anti-apoptotic, anti-oxidative, and anti-fibrotic role in several pathologies [77, 79, 80]. The evidence of a remodelling of S1PR expression in both OMA and DIE highlights S1PRs as potential endometriosis pharmacologic targets, and supplies a possible further application for FTY720 (fingolimod)-based therapies.

P-selectin is a hallmark of platelet activation: it is expressed on the cell surface when they get activated [81]. Its ligand P-selectin glycoprotein ligand-1 (PSGL-1) is expressed on the cell surface of most leukocytes and facilitates inflammation, haemostasis, thrombosis, etc. Recombinant P-selectin-Fc has shown to result in suppression of platelet aggregation, reduced angiogenesis and macrophage infiltration, decreased lesion size, FMT as reflected by reducing immunoreactivity to both αSMA and Col-I, and reduced fibrosis [81].

Relaxin (RLX)-2 is produced by the corpus luteum and placenta and is recognized for its potential effectiveness in fibrotic diseases of the heart, lungs, kidneys, and bladder. A study of LGR-7, a primary receptor of RLX-2, has shown that RLX-2 suppressed collagen-I, PAI-1, and IL-8 expression in ESCs [82] and all these molecules have an important role in fibrosis formation [83, 84]. LGR-7 increased cAMP production that activates PKA; it has been found that PKA decreased the phosphorylation of p38MAPK in ESCs [82]. p38 MAPK is essential in the pathophysiology of endometriosis by enhancing IL-8 production and improving cell proliferation in ESCs.

Therefore, the mechanisms responsible for the onset of fibrosis in various forms of endometriosis may be considered strengths to lead to new potential targets for new therapy for endometriosis.

## Data Availability

Not applicable.
